# Inhibition of Demineralization at Restoration Margins of Z100 and Tetric EvoCeram Bulk Fill in Dentin and Enamel

**DOI:** 10.3390/bioengineering6020036

**Published:** 2019-04-28

**Authors:** Cristina Leon-Pineda, Kevin Donly

**Affiliations:** Department of Developmental Dentistry, School of Dentistry, UT Health San Antonio, 7703 Floyd Curl Drive, San Antonio, TX 78229, USA; leonpineda@livemail.uthscsa.edu

**Keywords:** resin-based composite, restorative, bioactive, fluoride, remineralization

## Abstract

Recurrent caries is still considered the main reason restorations need to be replaced. There are different materials available now that promise to reduce the possibility of recurrent caries by releasing fluoride and inhibiting restoration marginal caries. The purpose of this in vitro study was to evaluate the demineralization inhibition potential of a non-fluoride-releasing resin (Z100^TM^ 3M, St. Paul, MN, USA) and a glass containing resin-based composite (Tetric EvoCeram Bulk Fill, Ivoclar/Vivadent AG, Schaan, Liechtenstein), which contains fluoride. Class V preparations were placed on 22 premolars; the gingival margin was below the cementoenamel junction and the occlusal margin was placed above the cemento-enamel junction. Ten teeth were randomly selected to be restored with Z100 while the other 10 were restored with Tetric EvoCeram Bulk Fill. Both groups were restored following manufacturer’s instructions. All teeth had an acid resistant varnish placed within one millimeter of the preparation margins. Both groups were placed in artificial caries challenge solution (pH 4.4). At the end of the 4 days; 100 µm buccolingual sections were obtained for each tooth; these were photographed under polarized light microscopy and the demineralized areas adjacent to the restorations were measured and quantified. The mean (±S.D.) area (µm^2^) of demineralization from the occlusal margin (enamel) and dentin margin were: Z100 2781.889 ± 1045.213; 3960.455 ± 705.964 and for Tetric EvoCeram Bulk Fill 1541.545 ± 1167.027; 3027.600 ± 512.078. Student’s t-test indicated that there was significantly less enamel and dentin demineralization adjacent to Tetric EvoCeram Bulk Fill compared to Z100; there was significantly less demineralization in enamel compared to dentin in both Tetric EvoCeral Bulk Fill and Z100. Tetric EvoCeram Bulk Fill performed better inhibiting demineralization at restoration margins when compared to Z100 and provided better demineralization inhibition in enamel than cementum/dentin.

## 1. Introduction

Dental caries is still an ongoing problem not only for adults but also for children. Approximately 59 percent of children between the ages of 12 and 19 years old have experienced dental caries [1]. Although there are different restorative options for dental caries, patients and their parents are leaning towards more esthetic options. While resin-based composites provide excellent esthetic results, we continue to encounter recurrent caries. Recurrent caries is considered the main reason restorations need to be replaced, regardless of restorative material [2]. Some of the problems encountered with resin-based composites that can contribute to recurrent caries are polymerization shrinkage, microleakage, technique sensitivity, sealing capacity, biodegradation of the interface, and mechanical degradation of the interface [3].

There are different materials available now that promise to reduce the possibility of recurrent caries by releasing fluoride and inhibiting restoration marginal caries [4]. These materials contain bioactive glass which has been shown to have an antimicrobial effect on oral bacteria. [5]. The antimicrobial effect of the bioactive glass is attributed to the release of calcium and phosphate ions which have a toxic effect on bacteria. As a result, there is neutralization of intraoral pH. Other currently available restorative materials release fluoride, but have not been shown to inhibit tooth demineralization [4]. Currently, there is no information in the literature that indicates this buffing effect and release of calcium and fluoride will inhibit demineralization of adjacent tooth structure. 

The purpose of this in vitro study was to evaluate the demineralization inhibition potential of non-fluoride-releasing resin (Z100^TM^ 3M ESPE, St. Paul, MN, USA) and a glass containing resin-based composite (Tetric EvoCeram Bulk Fill, Ivoclar/Vivadent AG, Schaan, Liechtenstein) which contains fluoride. 

## 2. Materials and Methods 

For this in vitro study, we collected 22 extracted permanent teeth from the University of Texas Health Science Center at San Antonio (UTHSCSA) Pediatric Dentistry Clinic. Currently, UTHSCSA has a standing Institutional Review Board (IRB) acceptance of using human teeth for studies without individual IRB review for each study because extracted teeth were accumulated with no identifying features. The teeth were stored in deionized water until the initiation of the experiment to avoid dehydration. A class V preparation was placed on the buccal surface of each tooth with the occlusal margin in enamel and the gingival margin in dentin/cementum, which provided a restoration where caries inhibition in enamel and dentin would be easily evaluated on sections through the tooth. [6]. An acid resistant varnish was placed on each tooth within one millimeter of the preparation margin. Ten teeth were selected randomly and restored with non-fluoridated resin (Z100), which acted as the control, and the other 10 teeth were restored with the fluoride containing resin-based composite (Tetric EvoCeram Bulk Fill). For the Z100 group Scotchbond Multipurpose Adhesive (3M, St. Paul, MN, USA) was used as bonding agent and for the Tetric group, Adhese Universal Bonding Agent (Ivoclar/Vivadent AG, Schaan, Liechtenstein) was used. 

After the teeth were restored, they were placed in an artificial caries solution (pH 4.4) for four days [7]. The artificial caries solution consisted of 2.2 mM Ca^+2^, 2.2 mM PO_4_^−3^, 50 mM acetic acid [7]. At the end of the four days, the teeth were sectioned buccolingually, going through teeth and restorations, using a Silverstone/Taylor hard tissue microtome (Scientific Fabrications, Colorado Springs, CO, USA). 100 µm sections were obtained from each tooth. Each section was then photographed using polarized light microscopy in an imbibition media of water [6]. A computerized imaging system (Image Pro, Media Cybernetics, Rockville, MD, USA) was used to measure the lesions 100 µm adjacent to the enamel and dentin/cementum restoration margins [6]. 

## 3. Results

The mean (±S.D.) area (µm^2^) of demineralization from the occlusal margin (enamel) for Z100 was 2781.889 ± 1045.213 and for Tetric EvoCeram Bulk Fill was 1541.545 ± 1167.027 (Table 1). The mean (±S.D.) area (µm^2^) of demineralization from the gingival margin (dentin/cementum) for Z100 was 3960.455 ± 705.964 and for Tetric EvoCeram Bulk Fill was 3027.600 ± 512.078 (Table 1). A Student’s t-test for the different subgroups indicated we had significantly less enamel demineralization adjacent to Tetric EvoCeram when compared to Z100 (*p* = 0.023). When comparing dentin demineralization, a Student’s t-test indicated there was significantly less dentin demineralization adjacent to Tetric EvoCeram Bulk Fill, compared to Z100 (*p* = 0.003) and when comparing demineralization adjacent to Tetric EvoCeram Bulk Fill, there was significantly less demineralization in enamel compared to dentin (*p* = 0.001). Lastly, and when comparing demineralization adjacent to Z100, there was significantly less demineralization in enamel compared to dentin (*p* = 0.008).

## 4. Discussion

**Figure 1:**
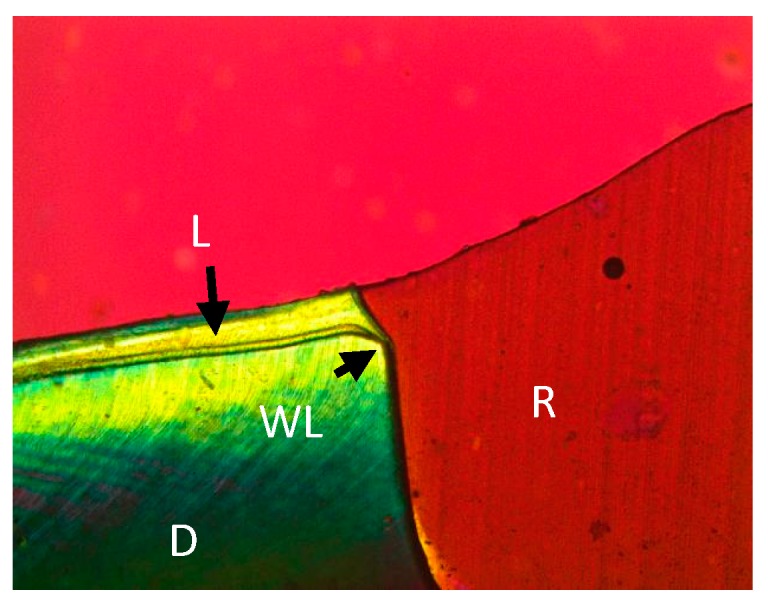
Z100 restoration in dentin at 10 times magnification (R—restoration, D—dentin, L—lesion, WL—wall lesion).

**Figure 2:**
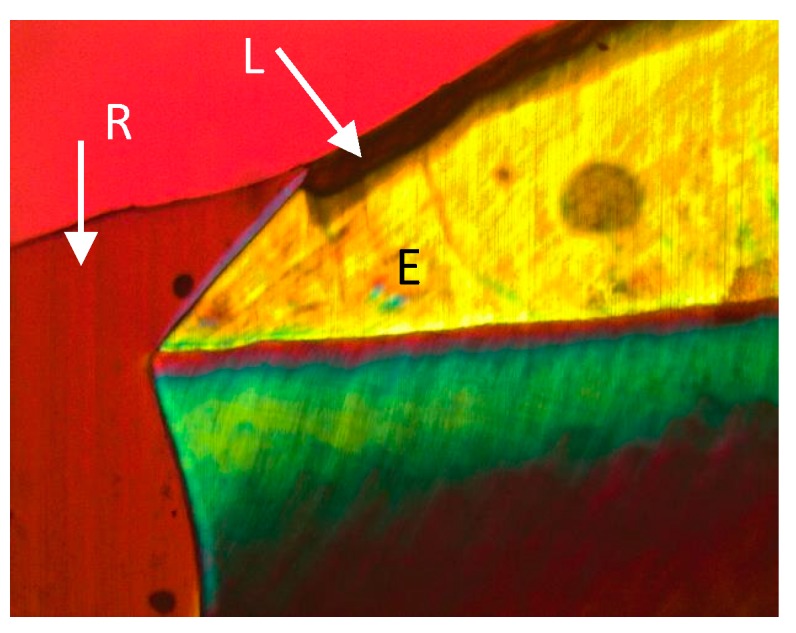
Z100 restoration in enamel at 10 times magnification (E—enamel, L—lesion, R—restoration).

**Figure 3:**
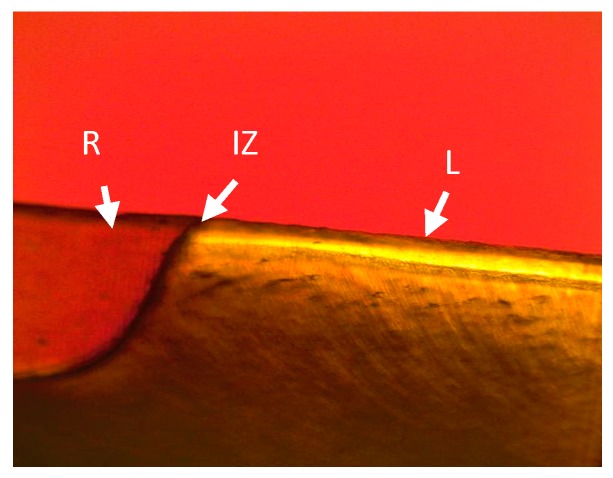
Tetric Evoceram Bulk Fill in dentin at 10 times magnification (R—restoration, L—lesion, IZ—inhibition zone).

**Figure 4:**
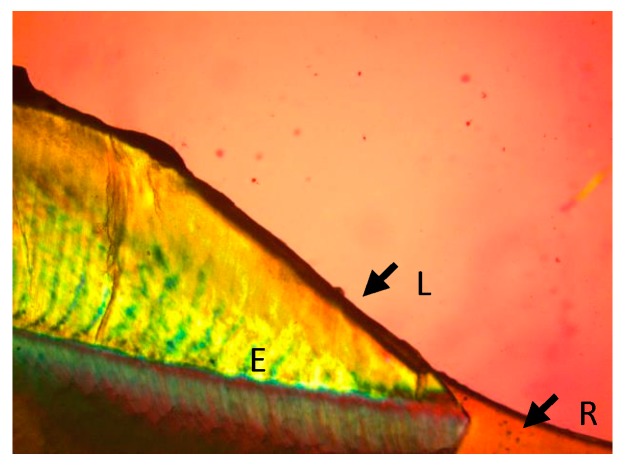
Tetric Evoceram Bulk Fill in enamel at 10 times magnification (E—enamel, L—lesion, R—restoration).

This in vitro study intended to examine the inhibition of demineralization at the restoration margins of two different restorative products. In this study, Tetric EvoCeram Bulk Fill performed better than Z100 inhibiting adjacent tooth demineralization in both enamel and dentin (Figure 1, Figure 2, Figure 3 and Figure 4). The Tetric EvoCeram Bulk fill had a similar appearance in demineralization inhibition as other fluoridated resins in previously completed studies [6,8,9]. This is most likely associated with less fluoride release from Z-100 resin composite when compared to fluoride containing materials [4]. When comparing the demineralization inhibition of Tetric EvoCeram Bulk Fill on enamel versus dentin/cementum, the performance was better in enamel than dentin/cementum. This is a reasonable finding since enamel is more resistant to acid attacks when compared to dentin due to its microstructure [8]. Actual inhibition zones (Figure 3) were found in 45% of enamel and 40% of dentin specimens in the Tetric EvoCeram Bulk Fill group, but none were found in the Z100 group. 

Although the effects fluoride has in caries prevention are widely known, the study of bioactive materials and their inhibition of adjacent tooth demineralization, has not received attention. The study of demineralization inhibition with fluoride containing materials started in the 1990s and has continued until the present day. Donly and Grandgenett [10] studied dentin demineralization with three different materials—two compomers and one glass ionomer material. In this specific study it was found that the glass ionomer material produced inhibition zones in dentin and enamel while no inhibition zones were found on the teeth restored with compomers [10]. This polarized light microscopy evaluation model has become an accepted technique for the evaluation of demineralization inhibition adjacent to fluoride-releasing materials [11]. 

Different studies have shown the ability fluoride containing restorations have in preventing wall lesions and in some cases create inhibition zones [6,12]. The incorporation of CaF_2_ nanoparticles into resins have shown similar fluoride release rates to those of resin-modified glass ionomers [13]. Nanoparticles of amorphous calcium phosphate (NACP) in nanocomposites have potential to help prevent or even delay the development of recurrent caries, which is considered the main reason restorations fail. 

Bioactive materials are not new, they have been around since 2010. With the continuous improvement of restorative materials, better clinical long-term results can be expected. We need to keep in mind that caries is an active process and for it to start and advance, there must be an imbalance between demineralization and remineralization [14]. Therefore, more clinical studies are needed to evaluate the true effect the oral biome has in calcium and fluoride releasing restorative products. In this particular study, very promising results were obtained with the use of Tetric EvoCeram Bulk Fill, including demineralization inhibition areas adjacent to the restorations. The main limitation of this research is that the study has been done in vitro and we may have different outcomes in the oral cavity. More research is needed to know the results intraorally with saliva, bacteria and constant acid challenges. 

## 5. Conclusions

Tetric EvoCeram Bulk Fill performed better inhibiting demineralization at the restoration margins when compared to Z100 and provided better demineralization inhibition in enamel than in cementum/dentin. 

## Figures and Tables

**Table 1:** Measurements of carious lesion areas in squared micrometers (µm^2^).

Enamel Tetric	Enamel Z100	Dentin Tetric	Dentin Z100
0.0000	4541.0000	3918.0000	4567.0000
1692.0000	1710.0000	3233.0000	3347.0000
1576.0000	3323.0000	3429.0000	3179.0000
2164.000	1733.0000	2444.0000	5270.0000
3766.0000	3418.0000	2663.0000	3662.0000
0.0000	3740.0000	2984.0000	3786.0000
2465.0000	2879.0000	2653.0000	4923.0000
2043.0000	1950.0000	2524.0000	4273.0000
1546.0000	1743.0000	2753.0000	3201.0000
1705.0000		3675.0000	3547.0000
0.0000			3810.0000
